# Privacy regulations and economic efficiency: a dynamic perspective

**DOI:** 10.12688/openreseurope.16103.1

**Published:** 2023-08-31

**Authors:** Francesc Dilme

**Affiliations:** 1 University of Bonn, Bonn, North Rhine-Westphalia, Germany

**Keywords:** Privacy regulations, dynamic pricing

## Abstract

There are important public debates on how—or even whether—to regulate information about previous transactions in different markets. Examples are the regulation of cookies in online markets, secrecy laws in financial markets, and transparency directives in government purchases. In these debates, considerations of economic efficiency and fairness are central. Do more lenient information policies increase market efficiency? Do internet users benefit from cookies? Does more secrecy increase trade volume in financial markets? Does transparency on previous agreements deteriorate the bargaining power of public agencies?

This essay sheds light on the answers to these questions using insights from economic theory. We do so by analyzing a simple, stylized setting where the incentives of different agents in the market will be clear. Our focus is on the dynamic effects of information regulations, as a trader's incentive to reveal or conceal information is driven by its future use by other traders. We use our setting to assess the implications of information regulations on behavior, trade efficiency, or social welfare.

## Plain language summary

There are important public debates on how—or even whether—to regulate information about previous transactions in different markets. Examples are the regulation of cookies in online markets, secrecy laws in financial markets, and transparency directives in government purchases. In these debates, considerations of economic efficiency and fairness are central. Do more lenient information policies increase market efficiency? Do internet users benefit from cookies? Does more secrecy increase trade volume in financial markets? Does transparency on previous agreements deteriorate the bargaining power of public agencies?

This essay sheds light on the answers to these questions using insights from economic theory. We do so by analyzing a simple, stylized setting where the incentives of different agents in the market will be clear. Our focus is on the dynamic effects of information regulations, as a trader’s incentive to reveal or conceal information is driven by its future use by other traders. We use our setting to assess the implications of information regulations on behavior, trade efficiency, or social welfare.

## A simple setting

In line with the methods used in economic theory, we will discuss the working of the markets of interest through the following stylized, simple model. A market has many *sellers* and *buyers*, who randomly meet over time. In the market, each buyer (“she”) wants to purchases different *goods* over time, and knows her *willingness to pay* for goods. When a buyer meets a seller, the seller obtains some *information* (to be described below) about the buyer’s previous transactions. The seller offers a *price*, which is either accepted or rejected by the buyer.

In online markets, the buyer represents a typical Internet user, her private information is her wealth or alternative purchasing options, and the imperfect information online sellers have about previous transactions comes from the cookies in the internet browser. In financial markets, the buyer represents a typical trader who has private information through her own research or contacts, while the imperfect information that other traders have comes from word of mouth or financial reports. Finally, in the market for procurement of public goods, the buyer represents a government agency, her private information corresponds to budgetary constraints or superior knowledge of future tax revenue, and the imperfect information that service providers have comes from publicly disclosed (partial) information or observation about its implementation.

It is important to note that debates on information regulations in the aforementioned markets involve other topics beyond economic efficiency. For instance, in online markets, the right to privacy is often used as an argument in favor of more restrictive policies. In financial markets, secrecy about transactions is often seen as facilitating market manipulation or tax evasion. In markets for the procurement of public goods, transparency is often seen as a mechanism to prevent corruption and foster equality of opportunity. These considerations are not completely orthogonal to market efficiency and can be studied in addition to the purely economic considerations studied here.

### Related literature

Understanding the effects of transparency and secrecy regulations is important to the study of markets. Seminal works include Stigler (1961) on price observability, Arrow (1962) on innovation, Akerlof (1970) on asymmetric information, Spence (1973) on signaling, and Grossman (1981) and Milgrom (1981) on disclosure. ^1^ The common view among economists is that while more information often (but not always) enhances market efficiency, the impact on buyer and seller welfare is context-specific.

Previous work has mainly focussed on the role of information in static models, where buyers and sellers interact only once. In a static model, a monopolist always benefits from more information since he can use this information to price discriminate. ^2^ More information leads to more efficiency because, when a monopolist can price discriminate, he increases sales (see initial works by Stigler, 1980, and Posner, 1981, and Tirole, 1988, for a textbook treatment). More information also typically decreases buyer surplus, because the monopolist can better adjust the price to her willingness to pay. Nevertheless, recent literature has shown that buyers can benefit if carefully-chosen information is about them released (see Bergemann *et al.*, 2015, for example).

The study of the dynamic effects of information in markets with repeated purchases is much less developed. Most of the literature on bargaining with one-sided offers studies the purchase of a single good by a buyer facing one or more sellers. Its most influential result is the so-called *Coase conjecture* ( Coase, 1972; see Gul *et al.*, 1986, for a formal proof), which states that the price offered by a monopolist converges to the competitive price as offers become more frequent. Kaya and Liu (2015) verify that this result extends to the case where a buyer receives offers from a sequence of short-lived sellers, independently of the observability of the previous price offers. Some work has shown that the Coase conjecture fails under other assumptions, such as adverse selection ( Deneckere & Liang, 2006; Hörner & Vieille, 2009, and Daley & Green, 2020), capacity choice ( McAfee & Wiseman, 2008), or outside options ( Board & Pycia, 2014).

While the extreme information structures (full information and no information) are straightforward (see below), studying more realistic markets with imperfect information is much more involved, as each transaction is stochastically affected by the previous interactions and stochastically affects the future ones. The analysis of such markets requires combining recent developments in dynamic signaling (e.g., Dilmé, 2019) with recent developments in repeated bargaining (e.g., Lee & Liu, 2013). Kaya & Roy (2020), Kaya & Roy (2022) show that, in the presence of adverse selection, an upper bound on the buyer’s payoff when offers are private is lower than his payoff in some equilibria when offers are public (they consider a sequence of sellers), and also analyze the effect of increasing competition. This essay focuses on the recent developments in the analysis of information in dynamic markets, particularly on Dilmé (2022).

## Analysis

### Two extreme scenarios

We will first analyze two extreme scenarios. In the first scenario, sellers have no information about previous transactions, similarly to markets with brick-and-mortar shops where sellers cannot monitor the previous behavior of an individual buyer. In the second scenario, sellers have perfect information about the previous transactions of the buyer, which corresponds to an extreme version of transparency law where all details of each agreement are disclosed. Even though these benchmarks are not adequate for studying information regulations, they will help understand the case where some but not all information is available, which is the one with more practical relevance.

**No information:** Consider first the case where each seller does not have any information about the previous transactions of the buyer. In this case, each interaction is akin to the standard static monopolist seller, where the buyer meets only one seller. This is so because each seller’s behavior does not depend on the previous encounters, as he does not observe them. Furthermore, the buyer does not have “signaling motives”; that is, because her current decision will not affect the behavior of future sellers, she does not have the incentive to reject offers to signal that her willingness to pay is low. Hence, as in a static model, the buyer accepts any price below her willingness to pay and rejects all prices above it. It follows that each seller offers the monopolist price, which maximizes the expected profit from the sale.

Like a static monopolist, each seller sets an inefficiently high price. Hence, in the absence of information about previous transactions of the buyer, trade volume is suboptimally low.

**Perfect information:** The polar opposite case of no information is where sellers have perfect information about previous transactions. In this case, each seller observes which price each previous seller offered and whether the buyer accepted the price. Basic reasoning would dictate that information about previous trades should make the buyer should be worse off, since sellers have easier access to information that is potentially informative of her willingness to pay, hence they can tailor their prices accordingly. Nevertheless, anticipating rejection is observed by future sellers, the buyer may have the incentive to reject a given offer, even if the price is lower than her willingness to pay. By rejecting the price, the buyer may hope that future sellers will believe she has a low willingness to pay and hence will set low prices to ensure trade. Dilmé (2022) shows that this is the case: prices are low and the buyer’s payoff is maximal under perfect information.

Paradoxically, when the information about the previous transactions is perfect, the information that these transactions contain about the buyer’s willingness to pay is minimal. Each seller offers a low price, which is accepted by the buyer independently of their willingness to pay, making the acceptance decision not informative.

### Intermediate information

The previous observations indicate that, while no information leads to inefficient high prices, perfect information about previous purchases makes the market efficient and gives the buyer part of the trade surplus. In practice, perfect information is not feasible, both because of limits in the tracking technology and because of privacy or confidentiality considerations. For example, cookies are typically unable to perfectly monitor the user’s purchase history (users can use different browsers or devices or use “incognito” modes that deactivate cookies). Similarly, traders or public institutions may not be able to disclose some details of the terms of trade in the previous transactions because of confidentiality clauses (necessary to preserve sensitive information) or to preserve privacy rights.

We follow Dilmé (2022) by introducing imperfect information to our model while abstracting from the fine details of how imperfect information is generated in practice. We then assume that each buyer’s interaction with a seller generates a “signal”, a random piece of “imperfect” information about the price and acceptance decision. Sellers observe the signals generated by the previous transactions and hence can partially infer the previous behavior by the buyer. A more informative signal conveys more precise information (e.g., through better tracking systems, transparency laws, or legislation restricting secrecy).

We begin by observing that extreme prices (very low or very high) tend not to generate much information about the buyer’s willingness to pay. Intuitively, if a seller offers a low price (that is, one that the buyer accepts it with a high probability regardless of her willingness to pay), acceptance is likely and uninformative. Similarly, rejection is likely and uninformative if a seller offers a high price. Only intermediate prices (those are accepted only when the buyer has a high enough willingness to pay) generate significant information, as both acceptance and rejection are likely and informative.

The previous observation implies that the more informative the signal about previous transactions is, the less likely the sellers will be to offer intermediate prices. The intuition is as follows. The rejection of an intermediate price leads future sellers to believe that the buyer has a low willingness to pay, inducing them to offer low prices. Hence, if an intermediate price is offered, the buyer has a strong incentive to reject it, even if his willingness to pay is high: by forgoing part of the current surplus from trade, she secures a high surplus in future transactions. When, instead, the price is low, rejection is very unlikely, so a signal indicating rejection will tend to be disregarded as noise. Similarly, when the price is high, rejection is very likely, so a signal indicating acceptance will tend to be disregarded as noise as well.

Dilmé (2022) shows that more informative signals lead to more extreme prices. He analyzes the effect of more lenient information legislation by analyzing how more frequent extreme prices affect efficiency and welfare. The total effect of more lenient information regulation regarding previous transactions tends to be beneficial for buyers and efficiency, as the increase in the frequency of low prices more than compensates the fact that high prices are more frequent too. When instead regulation becomes more lenient information regulation regarding previous price offers, buyer welfare and efficiency may suffer. Intuitively, when prices are concealed, when a seller offers a low price and the buyer accepts it, future sellers may believe that a high price was offered and the buyer accepted it, hence assign a higher likelihood to the buyer having a high willingness to pay. The implication is that low prices are less attractive to buyers, hence sellers may have to further lower them to ensure trade.

The previous insights provide guidance for the evaluation of information policies and their effects on pricing, efficiency, and welfare. First, more lenient information regulations tend to be good for buyers. The reason is that more information strengthens buyers’ signaling motives, and so tends to induce sellers to offer lower prices to avoid rejection. Second, the effect of the policy may depend on which type of information it concerns. More information about trade volume tend to benefit buyers, but more information about previous prices may hurt them. Calculating the extent of these effects requires using adequate economic models and obtaining data to calibrate them.

## Conclusions

Understanding the effects of regulating information on fairness and efficiency is crucial to properly design regulations of cookies in online markets, secrecy laws in financial markets, and transparency directives in government purchases. This essay contributes by presenting recent developments in the economic theory literature.

We discussed a simple model that illustrates how changing the quality of the available information affects the market outcome. We have argued that increasing the available information increases the likelihood of extreme prices, which affect efficiency and welfare in opposite directions. We hope that the proper analysis of these economic forces, as well as legal or moral considerations, will guide the design of future information policies.

## Ethics and consent

Ethical approval and consent were not required

## Data Availability

No data are associated with this article
