# Supplementary material for: Privacy regulations and economic efficiency: a dynamic perspective
Source: Open Res Eur. 2023 Aug 31;3:131. [Version 1] doi: 10.12688/openreseurope.16103.1 (PMC10509602; doi:10.12688/openreseurope.16103.1)
Supplement: Peer review history

# Reviewer report: Reviewer response for version 1

**DOI:** 10.21956/openreseurope.17386.r34888

**Published:** 2023-09-19

**Authors:** Kyungmin Kim

**Affiliations:** 1 Emory University, Atlanta, Georgia, USA

This essay considers a problem that is not only of great theoretical interest, but also increasingly relevant in our daily lives. By now, we know that a lot of information about each of us is out there and many firms actively utilize such information to reach (or exploit) us. Certainly, there is abundant research on privacy (information) regulations. The author’s work stands out in that it analyzes the effects of information regulations in a fully dynamic and general setting; as the author well illustrates, most existing studies on privacy regulations consider a simple statis or two-period setting or a few stylized information regimes, each of which can be enough to study some basic effects of information regulations but cannot be used to think about optimal regulations.

 The result (insight) that is particularly important/intriguing is that better information about past transactions induces sellers to offer more extreme prices (low or high, not intermediate). This makes a lot of sense after reaching this essay, but it was far from expected. This is a very interesting theoretical result by itself, but it also provides some clear and deep testable policy implications.

 I thought that more information (less privacy) can be beneficial to consumers only when firms can use consumer information to customize their products to better suit consumers’ idiosyncratic needs. This essay taught me that the channel is not necessary; more information can be beneficial to consumers only through its impact on consumers’ strategic signaling incentives and firms’ reactions to them. This is a very nice, informative piece of writing.

Is the argument persuasive and supported by appropriate evidence?

Yes

Is the work clearly and cogently presented?

Yes

Is the topic of the essay discussed accurately in the context of the current literature?

Yes

Does the essay contribute to the cultural, historical, social understanding of the field?

Yes

Reviewer Expertise:

Information economics, search theory

I confirm that I have read this submission and believe that I have an appropriate level of expertise to confirm that it is of an acceptable scientific standard.

# Reviewer report: Reviewer response for version 1

**DOI:** 10.21956/openreseurope.17386.r34886

**Published:** 2023-09-05

**Authors:** Fei Li

**Affiliations:** 1 Economics Department, The University of North Carolina at Chapel Hill, Chapel Hill, North Carolina, USA

The study you've presented is a thought-provoking exploration of the complex interactions between information regulations and market dynamics. It effectively highlights the central role of economic efficiency and fairness in the ongoing debates surrounding various information policies in different markets. The essay's focus on analyzing a simplified setting with clear agent incentives contributes to a better understanding of the dynamic effects of these regulations. Overall, the study provides valuable insights into the implications of information regulations on behaviors, trade efficiency, and social welfare.

 Strengths:

1. **Clear Articulation of Central Questions:** The essay effectively outlines the key questions related to different information regulations and their impact on market efficiency and fairness. This provides a solid foundation for the subsequent analysis.
2. **Application of Economic Theory:** The study's use of economic theory to analyze the potential outcomes of lenient and restrictive information policies is commendable. It allows for a systematic examination of the incentives and behaviors of different market participants.
3. **Balanced Evaluation of Trade-offs:** The essay acknowledges the trade-offs associated with each type of information regulation, such as the balance between targeted advertising and user privacy in online markets or the trade-off between transparency and bargaining power in government purchases. This demonstrates a nuanced understanding of the complexities involved.
4. **Dynamic Perspective on Information Use:** By focusing on the dynamic aspects of information use and how traders anticipate the future use of information, the study offers a fresh angle to understanding the rationale behind revealing or concealing information.

Suggestions for Improvement:

1. **Empirical Validation:** While the study provides a valuable theoretical analysis, incorporating real-world data or case studies could strengthen its findings. Empirical evidence would help validate the theoretical insights and provide a more practical perspective on the impacts of different information policies.
2. **Consideration of External Factors:** The essay primarily focuses on the effects of information regulations within a simplified setting. However, real-world markets are influenced by a myriad of external factors such as cultural norms, technological advancements, and regulatory environments. Acknowledging these external factors could provide a more comprehensive understanding of the complexities involved in information regulation.
3. **Ethical Considerations:** Given the ethical implications of information policies, it would be beneficial to address the ethical aspects associated with the regulation of information. Exploring the ethical dimensions could enhance the essay's depth and relevance.
4. **Broader Context:** While the essay focuses on specific cases like cookies, financial secrecy, and government transparency, it could be valuable to broaden the discussion to include other relevant examples. This would help showcase a wider range of scenarios where information regulations play a crucial role.

Unassessed Aspects: The study's framework and findings are well-presented and discussed within the context of the essay's scope. However, the essay's specific content and arguments beyond what you've provided have not been directly assessed. Any statistical analyses or specific conclusions derived from the analysis have not been evaluated, as they are not included in the text you've shared.

 In conclusion, the study provides an insightful exploration of the implications of information regulations on market dynamics, efficiency, and fairness. It effectively employs economic theory and a dynamic perspective to shed light on these intricate interactions. By considering empirical evidence, external factors, ethical considerations, and broadening the scope of examples, the study could enhance its depth and relevance, making its insights even more valuable to ongoing debates.

Is the argument persuasive and supported by appropriate evidence?

Yes

Is the work clearly and cogently presented?

Yes

Is the topic of the essay discussed accurately in the context of the current literature?

Yes

Does the essay contribute to the cultural, historical, social understanding of the field?

Yes

Reviewer Expertise:

Economic Theory

I confirm that I have read this submission and believe that I have an appropriate level of expertise to confirm that it is of an acceptable scientific standard.
